# Acceptability of the Dapivirine Vaginal Ring and Oral Truvada Among African Users in Late-Stage of Pregnancy

**DOI:** 10.1007/s10461-023-04203-z

**Published:** 2023-11-07

**Authors:** Elizabeth T. Montgomery, Imogen Hawley, Lee Fairlie, Katie Bunge, Florence Mathebula, Juliane Etima, Prisca Mutero, Linly Senyama, Ashley Mayo, Marie C. D. Stoner, Jeanna Piper, Ivan Balan, Ariane van der Straten

**Affiliations:** 1 Women’s Global Health Imperative, RTI International, 2150 Shattuck Avenue, Berkeley, CA 94104 USA; 2 Department of Epidemiology and Biostatistics, University of California, San Francisco, San Francisco, CA USA; 3 Wits Reproductive Health and HIV Institute, Faculty of Health Sciences, University of the Witwatersrand, Johannesburg, South Africa; 4 MMWRI, University of Pittsburgh, Pittsburgh, PA USA; 5 Makerere University - Johns Hopkins University (MU-JHU) Research Collaboration, Kampala, Uganda; 6 Clinical Trials Research Centre, University of Zimbabwe, Harare, Zimbabwe; 7 Johns Hopkins Project, College of Medicine, University of Malawi, Blantyre, Malawi; 8 FHI 360, Durham, NC USA; 9 NIAID, Bethesda, MD USA; 10 Department of Behavioral Science and Social Medicine, Florida State University College of Medicine, Tallahassee, FL USA; 11 ASTRA Consulting, Kensington, CA USA

**Keywords:** Pregnant women, Dapivirine ring, Oral PrEP, Malawi, Uganda, Zimbabwe, South Africa, HIV prevention

## Abstract

The Microbicide Trials Network 042 study (MTN-042/DELIVER) is a two-arm, randomized, open-label Phase 3b trial that is evaluating the safety, adherence, and acceptability of the monthly ring and daily oral PrEP among HIV-uninfected pregnant people in four African countries. This analysis focuses on acceptability data captured qualitatively from a subset (n = 48) of the 150 people in the first cohort of the trial who were enrolled in late-stage pregnancy at 36 to 38 weeks gestational age and followed until after delivery. Single IDIs were conducted by trained interviewers at each clinic site using a semi-structured guide. Data excerpts of key codes pertaining to acceptability, pregnancy, and maternal health were summarized, reviewed and interpreted by multinational analyst teams. Although the product use period was relatively short, the data suggested several acceptability findings that may directly translate to longer durations of product use in pregnancy. The first was the overarching maternal sentiment that being able to protect both oneself and their baby was highly valued. The second was the importance of counseling support from providers not only because participants used methods that might generate side effects, but because pregnancy itself is a period with its own set of side effects. The third was that, similar to non-pregnant participants in other trials, here study products were generally liked and described as easy to use. Concerns about ring and oral PrEP use could be addressed with provider counseling and support and should form an essential component rollout among pregnant people.

## Introduction

The risk of HIV acquisition may be increased during pregnancy due to both biological and behavioral changes, and incident infection during pregnancy can substantially increase the risk of vertical transmission of HIV [1–5]. Consequently, pregnant people in HIV endemic settings are an important population to reach with effective biomedical HIV prevention methods. To date, evidence on the acceptability of new HIV prevention methods during pregnancy are limited.

Clinical trials have evaluated the acceptability of, and adherence to, the *oral tenofovir disoproxil fumarate/emtricitabine (TDF/FTC)* tablet (“oral PrEP”) and the dapivirine vaginal ring (“the ring”) among non-pregnant reproductive-aged people assigned female at birth in Africa [4, 6–13]. Pregnant people were excluded from enrollment into efficacy trials of the ring, however people who became pregnant were followed off product and had no adverse pregnancy outcomes [14, 15]. Exposure data in pregnant and postpartum people for treatment as well as HIV prevention, and data from the rollout of oral PrEP, show the drug to be safe and effective in pregnant people [16–20]. Yet, while pregnant people are willing to initiate oral PrEP, early discontinuation during pregnancy is high, particularly in young people, and limited research exists to understand the barriers to PrEP persistence in this population [21, 22].

The ring received a positive opinion by the European Medicines Agency (EMA) in July 2020, and prequalification approval from the World Health Organization (WHO) in November 2020. In 2021, the WHO released updated consolidated guidelines for HIV prevention which included a recommendation for the ring as an HIV prevention choice for women at substantial risk of HIV infection [23]. The ring has since been registered for use in several African countries, and demonstration projects are planned for provision of the ring in selected public health clinic settings. It is important to consider how both the ring and oral PrEP adoption and use are influenced by acceptability considerations (e.g., intensity of systemic side effects), among pregnant people, particularly as they are also experiencing physical constraints and symptoms of pregnancy and concerns about their baby.

The Microbicide Trials Network 042 study (MTN-042/DELIVER) was a two-arm, randomized, open-label Phase 3b trial that is evaluating the safety, adherence, and acceptability of the monthly ring and daily oral PrEP among HIV-uninfected pregnant people in four African countries. This analysis used qualitative data from a subset of the first cohort of the trial where 150 women were enrolled in late stage of pregnancy and focuses on comprehensively assessing defined constructs of acceptability using an existing theoretical framework.

## Methods

### Study Design and Population

The MTN-042/DELIVER study was designed to enroll pregnant people in multiple cohorts defined by gestation period, starting with those at most advanced gestation, and moving to earlier gestation with each cohort [24]. Safety reviews were scheduled between each cohort before onward progression. Participants received HIV prevention and product adherence counseling at each study visit. Prior to enrollment, the currently known safety, effectiveness and regulatory approval status of the study products among non-pregnant women was described in the informed consent forms. Additionally, the informed consent forms and staff described the stepwise three-cohort study design and safety monitoring, specifying their role in the first group.

No deliveries occurred at any of the research sites, however participants had to be planning to deliver their babies at a public hospital or similar public health facility affiliated with the research clinic site where adequate medical records could be obtained.

During cohort 1, the multi-site trial enrolled 150 healthy, HIV-uninfected pregnant people in Blantyre, Malawi; Kampala, Uganda; Johannesburg, South Africa; and Chitungwiza, Zimbabwe between January 2020 and April 2021. Participants between the ages of 18 and 40 who had an uncomplicated singleton pregnancy were randomized to the ring or oral Truvada™ in a 2:1 ratio (ring: oral PrEP) between 36 and 38 weeks gestation. Sites worked cooperatively to reach the overall accrual goals. Depending on arm assignment, participants were instructed to use the ring continuously for approximately one month or until their pregnancy outcome, or to take one daily oral pill until their pregnancy outcome. Participants were on study product from randomization to discontinuation, for an average of 23.1 days (range: 1–45 days, median of 23 days). Participants using the ring were instructed to remove the ring when they believed they were going into labor and asked to return it to the research staff before study termination during the postpartum period.

### Qualitative Subsample

A secondary objective of the MTN-042 trial is to characterize acceptability of the ring and oral PrEP. For cohort 1, this was measured qualitatively among a subsample of 48 participants who were randomly selected to complete an in-depth interview (IDI). Participants were interviewed around 38 weeks gestation or a minimum of two weeks after study product dispensation, and before exiting the trial. Because the sample included people in late-stage pregnancy, several selected participants went into labor before their anticipated IDI date, and 17 IDIs (35%) were conducted postpartum. The qualitative subsample per site was proportionally aligned, by total number and arm assignment, with the total number of enrollments at that site (see Table 1). Single IDIs were conducted by trained interviewers at each clinic site using a semi-structured interview guide. Interviews were conducted face-to-face in private locations in the language of the participants’ choice (Chichewa, Luganda, Sesotho, isiZulu, Shona and/or English) and lasted an average of 73 min (range 36–113 min). IDIs explored study product acceptability and user experience, the focus of this analysis. Additional topics included but were not limited to: experiences while pregnant and with study participation overall; healthcare seeking during pregnancy; COVID-19; PrEP disclosure and community attitudes; sexual activity during pregnancy; and overall satisfaction with their assigned study products.

**Table 1:** Baseline demographic characteristics of participants enrolled in MTN-042/DELIVER cohort 1

	Overall (n = 150)	Qualitative sample (n = 48)
Age, median (interquartile range (IQR))	25.0 (21.0–28.0)	23.5 (21.0–26.5)
Product assignment, n (%)		
Oral PrEP	49 (32.7%)	15 (31.3%)
Vaginal Ring	101 (67.3%)	33 (68.8%)
Site, n (%)		
Blantyre, Malawi	27 (18.0%)	8 (16.7%)
Johannesburg, South Africa	42 (28.0%)	16 (33.3%)
Chitungwiza, Zimbabwe	37 (24.7%)	11 (22.9%)
Kampala, Uganda	44 (29.3%)	13 (27.1%)
Parity, n (%)		
0	52 (34.7%)	14 (29.2%)
1	53 (35.3%)	22 (45.8%)
> 1	45 (30.0%)	12 (25.0%)
Estimated gestational age, when enrolled, median (IQR)	36.3 (36.1–36.6)	36.3 (36.1–36.8)
Has primary partner, n (%)	146 (97.3%)	47 (97.9%)
Primary partner knows enrolled in study, n (%)^a^	123 (82.0%)	40 (83.3%)

^a^Denominator is number of participants with a primary partner

Signed informed consent forms approved by the relevant ethics committees were obtained for each IDI conducted. All IDIs were audio recorded and transcribed into English by study site staff or their internal or external designees using a one-step translation and transcription method, with transcripts reviewed for quality by interviewers and qualitative analysts.

### Qualitative Analysis

**Fig. 1:**
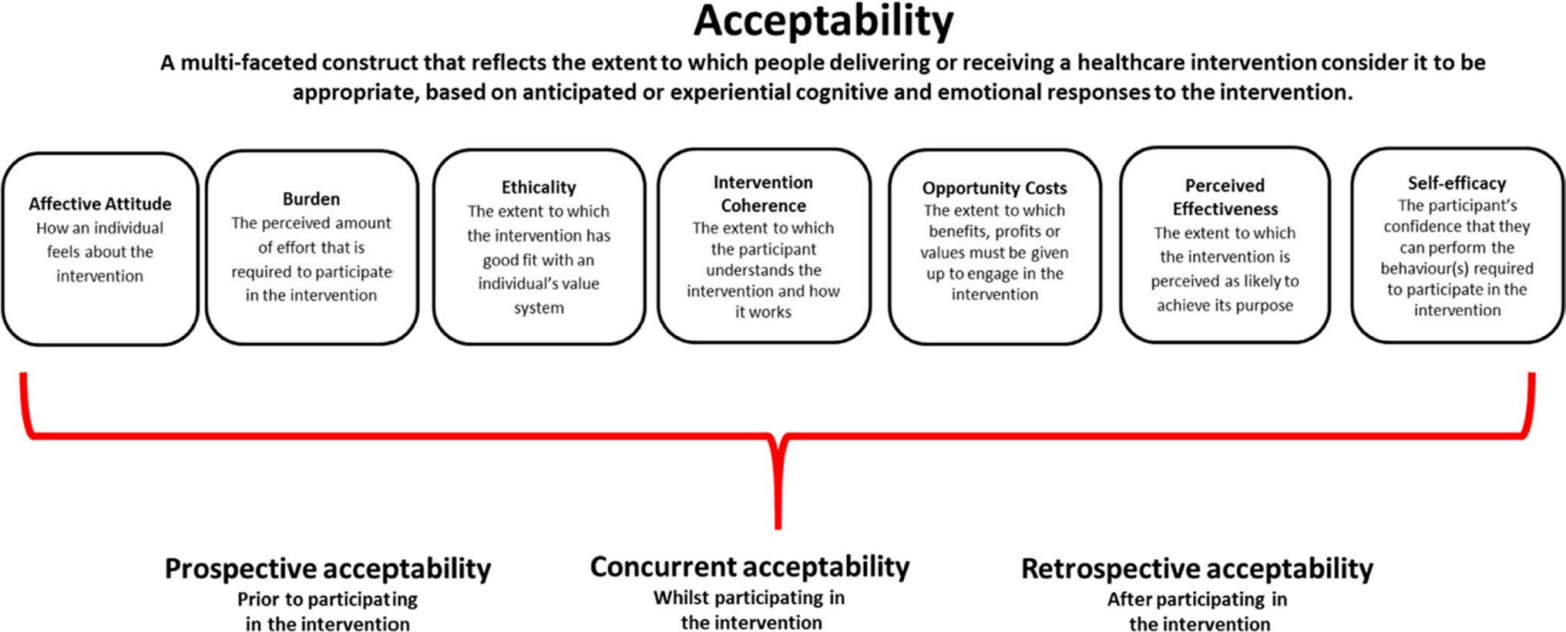
The Theoretical Framework of Acceptability comprising seven component constructs

An initial codebook was developed based on the trial’s research questions and the structured IDI guide. The people who participated in cohort 1 IDIs were predominantly still pregnant and assigned to study products at the time of the interviews, corresponding to “concurrent acceptability” (see Fig. 1), while the remainder were postpartum and expressing retrospective accounts of acceptability after having used their assigned PrEP methods.

The codebook for analysis included descriptive codes that directly corresponded to topical areas relevant to the study (e.g. ring, tablet, pregnancy, study product attributes, side effects). In addition, there were analytical codes that corresponded to the primary objective of exploring acceptability using the Theoretical Framework of Acceptability (TFA). The purpose of using the TFA for coding and analysis was to comprehensively examine aspects of the broad and multi-dimensional concept of “acceptability” for an intervention by delineating “acceptability” into smaller, defined constructs (e.g. affective attitude, burden, etc. as depicted in Fig. 1 [25]. This methodological approach provided greater confidence that exploration and interpretation of intervention acceptability considered a defined range of factors influencing attitudes. The TFA was previously used to assess prospective acceptability of the ring and oral PrEP among pregnant and lactating people during formative work at the same four sites in sub-Saharan Africa [25, 26]. Transcripts were uploaded into a qualitative software package (Dedoose; Los Angeles, CA: version 9.0.17) and coded by a team of four trained qualitative analysts in the United States. Weekly coding meetings were held over the course of approximately three months to review exported code reports to test intercoder reliability, reach consensus on the interpretation and application of codes, and refine the codebook iteratively as needed. Following the coding process, data excerpts of key codes pertaining to acceptability (descriptive codes and analytical codes encompassing all TFA constructs), pregnancy, and maternal health and were summarized thematically, and illustrative quotes were selected. Quotes and results interpretation was reviewed and vetted by local social science staff at each participating African site. Quantitative data on participant demographics was summarized in Stata version 17 (StataCorp 2021 College Station, TX). Gender identity was not an exclusionary criterion and was not collected in participant demographics, so we refer to all pregnant participants in gender neutral terms.

## Results

### Study Sample

The characteristics of the 48 participants in the qualitative subsample, compared to those for the entire cohort 1 of the MTN-042 sample, are presented in Table 1. Participants in the qualitative sample had a median age of 23.5 years and were representative of the parent study randomization schema and the site-based enrollment targets. The median gestational age at enrolment was 36.3 weeks, and 29% were nulliparous prior to delivery. The qualitative subsample and the overall sample were similar across characteristics, with the exception that a higher proportion of participants in the qualitative group had 1 prior delivery. Most participants had a primary partner, and over three-quarters reported that their partner knew about their study enrollment.

### Ring and Oral PrEP Acceptability

Data pertaining to the overall acceptability of the study products are described below, with presentation of how attitudes and experiences aligned with the seven constructs of the TFA framework (Fig. 1). Acceptability attitudes are presented in groups of two or three TFA constructs per group, based on how the findings fit together conceptually. For example, we first present findings about participants’ understanding of the purpose of the research intervention (TFA construct: intervention coherence) and how the study products were believed to “work” to prevent HIV infection (TFA construct: perceived effectiveness). These constructs include some of the foundational underpinnings of intervention acceptability that speak to background understanding and knowledge comprehension. Secondly, we present how the products aligned with participants’ personal beliefs, study values, and ethics (TFA construct: ethicality), as well as their sense of self-confidence and ability to implement preventive behaviors to use the products (TFA construct: self-efficacy). Both of these TFA constructs link to how acceptability is impacted by internal confidence and beliefs Lastly, the acceptability of the study products in terms of how participants described feeling about them (TFA construct: affective attitude), the effort they required to be used and side effects (TFA construct: burden) and what had to be given up to engage in product use (TFA construct: opportunity costs) are presented. This final group of acceptability constructs address intervention qualities that address somewhat more external, product- and delivery-oriented or pragmatic components of the intervention. Within each results section, findings that apply to both study products are presented in combination, or otherwise specified as ring- or oral PrEP-specific. Data for all sites is combined within each section both because there were no major differences across sites, and further, understanding variations by site was not an objective of the study and design.

### Intervention Coherence and Perceived Effectiveness: Knowledge and Understanding of Product Use

Overall, the participants understood the fundamental research premise that the study products protected them against HIV when they were used consistently and correctly, although a few had incorrect perceptions of how much longer they would be protected after product use was terminated at the onset of labor. Participants’ intervention coherence was facilitated by study staff explaining the research process, reminding them of the product’s function and emphasizing they were available for support. Participants stated that they trusted that the study staff would not conduct this research if there was potential for the product to cause harm. Additionally, participants described the important role that staff contributed to intervention delivery by 1) inserting the ring “more successfully” if that was needed or helping them place the ring so that it would work properly and feel comfortable; 2) diminishing anxiety around the success and safety of their delivery and baby following study product use; 3) facilitating transfer of product knowledge and approval to local public health care maternity units; and 4) facilitating knowledge about research trial stages that are driven by safety monitoring. A participant in Malawi summarized their understanding as follows:

It was because they [study clinic staff] were able to explain everything to me like ‘you are not the first one to use the ring. Studies with animals, then a smaller group of people then a bigger group then we reached to those people who were pregnant as well. So we would like to reach all stages of pregnancy in this research and we have started with you pregnant mothers who are eight months above then later we will approach those who are four or five months pregnant so that we should see what is going to happen.’ (Ring-user, Blantyre)

Participants who were assigned to oral PrEP expressed a preference for this method over the ring as they held the same initial worries as those assigned to the ring about potential problems (e.g. pain, impact on baby). As with ring users, worries about long-term health impacts of exposure to oral PrEP on the babies were expressed, but later eased by explanations from study staff. Other participants voiced uncertainty and mixed understanding (coherence) as to whether their baby would directly benefit from HIV prevention from oral PrEP.

Although most participants demonstrated good understanding of how the product works and is used, they were enrolled in a safety trial, and perhaps unsurprisingly maintained fears about potential biological or anatomical abnormalities that could occur, including that the ring could get stuck inside their reproductive systems, cause general harm to the baby, get lost or dissolve inside the body, fall out of the body, cause pain, or that the baby might get “tangled” with the ring during delivery. Additionally, several participants had apprehensions that the ring might cause the baby to acquire “abnormalities,” “disabilities” or experience developmental delays. Although less common, some pregnant participants reported a perception that the ring would block the natural process of giving birth: “I thought it would block [the womb] and my baby would not be able to come out” (*Ring-user, Johannesburg*). A participant in Uganda shared that they had concerns that the ring would bend and press against their organs, muscles and the baby while they were seated in a hard chair sewing for work.

In addition to understanding the purpose of these biomedical interventions to prevent HIV, almost all participants interviewed perceived the study products were effective. These feelings were reinforced by the health educational information they received, receipt of negative HIV test results throughout the study (despite its short duration), and interpretations of side effect experiences as evidence that a drug was working. Participants were confident that the study products protected their unborn babies as well as themselves. A few participants acknowledged that the protection from the study products was not fool-proof or absolute, yet they still felt generally safe from HIV. Alongside this, some participants emphasized that the study products offered protection only when used correctly. Overall, participants understood the necessity of adhering to their assigned study product for an entire month-long interval of use, with some explaining how the ring slowly releases medicine in the body. Although rare, some participants expressed doubt about whether the study products were effective, opting to wait and decide when they saw an HIV negative test result post-study.

Participants’ motivations to join the study and the aspect of acceptability defined by perceived effectiveness to prevent HIV were mentioned to friends, family, partners, and others, and this was particularly important because they wanted to ensure their safety and that of their child from any partner behavior:

…What I like most [about] prevention of HIV… even if he [partner] misbehaves [engages in sexual relations] out there it [HIV] will catch him alone. … Me and my child will be safe (Ring-user, Chitungwiza)

### Ethicality and Self-efficacy: Beliefs and User Interactions with Products

Participants using the ring overall believed in its benefit, and in particular how it protected their baby, and this suggested strong ethical alignment with the purpose of the research study. Participants who sought approval from their partners about study participation stated the partners had no problem with it, also citing their partner’s approval of protecting their baby’s wellbeing. When participants were asked about what they thought the broader community (non-familial) thought of the ring, opinions were divided, with a portion believing that the community perceived the ring and the research study overall as being “good”, while another portion had heard negative rumors circulating about the ring, the study, and study staff. Specifically in Zimbabwe, several claims about how the study and its staff are “evil” and were not to be trusted were cited, often in relation to the research practice of taking blood specimens as part of the study procedures:

Ha-a [no], I have not experienced it but there are some people who I heard saying that…they were saying, “he-e”…when the vehicle was passing by, [they were saying] “he-e” … “They are Satanists’ vehicles; they want people’s blood” and so forth. (Ring-user, Chitungwiza)

Participants seemed to easily brush aside these rumors because ultimately, their values about protecting their health and their baby’s health were aligned with and recognized by the research.

Several participants mentioned feeling confident in their self-efficacy and ability to use the ring or oral PrEP, as assigned, because either they liked knowing that they would prevent HIV or because they had committed to being in the study and were behaviorally motivated to use the product and prevent HIV. In regard to technical skills, there was a divide between ring users who felt confident in inserting/removing the ring on their own and those who felt they needed help from professional staff. Those who felt confident cited support from study staff, having early experiences of the ring insertion going well, and knowing that they needed to be self-sufficient with ring removal before labor. The participants below eloquently articulated the positive impact of gaining self-efficacy around ring use, and the confidence and pride that followed:

that fact that you did it yourself, you were just taught a new thing and you successfully did it alone-- It’s just fascinating… It’s like being asked to jab yourself an injection, and successfully do it, you will be happy that I have injected myself successfully, uhm.” (Ring-user, Chitungwiza)

... because when you stay with people for some time you develop the self-esteem driving away the fear. So, as I stayed with the service providers, they became my friends and had gone through for me and I became an expert [in using the ring].” (Ring-user, Kampala)

Those who were not confident in their ability to insert/remove the ring on their own (as touched on above) mentioned feeling less capable than the study staff who have ample experience with ring procedures, being nervous about not being able to remove the ring when it is deemed necessary during the “craze” of labor/delivery, and/or physically not being able to reach around their pregnant bellies.

Most participants who were assigned to oral PrEP reported no trouble taking the pills every day as they expressed a sense of accountability, an ethic, to being a part of the study as well as wanting to feel protected from HIV. A few oral PrEP users were less confident in being able to adhere to the daily regimen because it can become “*boring*” (tedious) to swallow the pills.

### Affective Attitudes, Burdens and Opportunity Costs: Attitudes Towards and Experiences with Product Use

As noted above, participants expressed pre-emptive concerns about the ring causing physical problems or pain. However, once they had tried it, participants’ affective attitudes towards the ring were positive across all research settings. A common description of ring use was that it felt “*normal*” in both physical and psychological ways, meaning that the ring didn’t interfere with their daily routine and caused no worry. A participant in Zimbabwe described the ring as a “condom” because it was unnoticeable, simple, as well as safe and trustworthy:

Haa, the ring, I was taking the ring [was] like a condom, aunt … A condom if you wear it-- You do not even feel that you have something inserted … I took it as something simple, you see … I was not thinking or being afraid of anything or what … I just knew that haa, there is no problem. (Ring-user, Chitungwiza)

Another frequent and favorable remark—perhaps because of their size in late-stage pregnancy—was that the ring did not interfere with walking for *most* participants. These participants also expressed liking the ring because of its simplicity and low physical and mental burden: they could leave it in place and forget about it, without feeling it further.

I was stressed about putting something [the ring] inside me because I thought I would not be able to walk or it would come out while I’m walking or fall out in the presence of other people. But so far everything is hundred percent fine. (Ring-user, Johannesburg)

Although infrequent, a few pregnant participants also reported a perception that the ring was causing feelings of heaviness in the vagina.

There were mixed attitudes about the ring’s impact on sex. Some thought the ring increased their sex drive, or improved sex because of added lubrication. Others disfavored the added wetness, described by one as “fluid ejection” that was *attributed to* ring use—and this was described as “disappointing” and “hard to get used to” by others.

Maybe that of fluid ejection, but it is not an everyday or usual thing. But when it came, I later got used to it. However, it is not an easy thing to get used to. (Ring-user, Kampala)

Other participants recognized and described that they were less interested in sex, but acknowledged that this was not because of the ring, but because of pregnancy.

Most participants noted that the study products were simple and straightforward to use, requiring minimal burden, and that reservations or concerns with ring use were dispelled with time. Some side effects were associated with ring use, including systemic side effects such as headache and nausea and local effects like itching and burning, or scratching:

During the first time I inserted that ring, I felt some itching, and I felt very hot in my stomach (Ring-user, Blantyre)

We were going to look for firewood. It [the ring] kind of scratched me, such that I saw some drops of blood on the pant. (Ring-user, Chitungwiza)

Participants remarked that some side effects that are generally common with pregnancy they had first attributed to the ring, such as feet-swelling, challenges with breathing, and these were overcome through counseling with research staff.

And I even explained to them [study clinic staff] about the problem of the swelling feet which I had, and they explained that it wasn’t really a problem, and I should not get worried with it because I will get better as soon as I deliver my baby. And they explained again that it wasn’t the ring which was making my feet to swell but it was normal for every pregnant woman to experience such things when she is on the last month… (Ring-user, Blantyre)

Oral PrEP users reported some negative attitudes and experiences of feeling “choked” by swallowing the pills, while in the case of the following participant, also appreciating the benefits and enduring the burdens:

Sometimes they [the tablets] choke me, health worker…. Yes, actually I don’t like them, as they say ‘too much’ of anything… but the fact that it protects and I am not the one who has bought it, I force myself to take them and don’t miss taking, I force myself to take them in time, I follow all instructions the health worker gave which help me as an individual.” (oral PrEP-user, Kampala)

Other participants remarked that oral PrEP contributed to pain associated with toothaches, gastro-intestinal discomfort, dizziness, rashes, nausea, effects on appetite. Both the burden and the convenience of oral PrEP being another of several oral medications already required for pregnancy was noted.

However, in balance, more participants highlighted the *lack* of burden associated with, or *lack* of effort required for using the ring or oral PrEP than those remarking upon burdens. Participants in general had positive attitudes towards the study products and found them to be a low burden.

Few opportunity costs were reported to be associated with ring and oral PrEP use. Indeed, opportunity costs were mentioned in two types of situations: The first type related to sex—a negative change in the desire for sex by the participant or their partner due to ring use. The other was a loss in comfort when visiting friends due to increased vaginal discharge. By contrast, another participant who also experienced increased vaginal discharge did not consider it to be a burden, inconvenience, or prompting a loss to their social activities.

## Discussion

This qualitative analysis from a subset of pregnant participants in the first cohort of the MTN 042 trial offers several important and novel insights into acceptability and use of prevention methods, particularly the ring, a new PrEP option, in late-stage pregnancy. The trial was designed to assess the safety and pharmacokinetics of the drugs in pregnant users and infants retroactively after use of the products. Although the product use period was relatively short, the data suggested several findings that may directly translate to longer durations of product use in pregnancy. Use of the TFA to comprehensively examine potentially important components of acceptability offered a wide range of attitudinal perspectives. The following three key findings emerged as the most common and salient factors contributing to women’s considerations of product acceptability. The first is that the overarching maternal benefit of being in a position to protect both themself and their baby was highly valued. The second was the importance of counseling support from providers not only because participants used methods that might generate side effects, but because pregnancy itself is a physically and emotionally vulnerable period that has its own set of “side effects”. The third and final key acceptability finding was that ring and oral PrEP were—in general—liked and described as easy to use, as they have been by non-pregnant participants in other trials.

Many acceptability attitudes were closely centered around beliefs of the impact—either perceived to be negative or positive—on the baby. It is intuitively logical that a person in the late stage of pregnancy would, literally, bear this sense of responsibility, and the evidence presented in this analysis helps reinforce the necessity of ensuring users’ knowledge about mechanism of action and product effectiveness for the baby, and once safety is established, mitigating concerns about potential harm. Historically there is a lack of data and research about the safety and effectiveness of HIV prevention products (and many other medicines) during and post pregnancy. Increasingly, there is attention towards redressing these research gaps—indeed, this MTN-042/DELIVER study [24] and MTN-043/B-PROTECTED [27] are at the vanguard of doing so for the ring and oral PrEP, HPTN 084 is following use of cabotegravir in pregnant participants, and Gilead Sciences has designed a current injectable Lenacapavir trial (www.purposestudies.com) to accommodate pregnant and lactating participants. As a wider berth of options for people of reproductive potential is made available, it is essential to address their knowledge and fears around how active agents may impact the baby, as a strategy to support safe, correct and consistent prevention method use. The participants in this research who had early or pre-emptive worries, consistently overcame them with actual use, education and clinical staff support.

Further, a higher proportion of participants in the MTN-042/DELIVER study may have disclosed study participation, ring or oral PrEP use, and had the support of partners, compared to their nonpregnant counterparts [3, 4, 28]. Pregnant people’s descriptions of partner attitudes towards the ring and oral PrEP, and the impact study products might have on sex were *not* widely noted as concerns. The difference between pregnant and non-pregnant populations regarding real or actual problems with PrEP disclosure and partner dynamics may be a consequence of the rationale for PrEP use being directed towards the baby’s wellbeing and health, instead of focusing on the adult sexual relationship. Further, the partner (in most cases) and the participant are both the parents of the unborn child, so it stands to reason that a pregnant person might be more likely to have wanted or needed their partner’s support and engagement before joining the research.

Another key finding from these data was the importance of clinical and counseling staff addressing pre-emptive and actual worries related to study product use, and, further, *disaggregating* experiences or side effects attributed to products that might regularly occur within pregnancy. Participants were concerned about being able to walk with a ring in situ, and some feared the active agent causing congenital anomalies or developmental disabilities. Others were concerned about challenges related to the physicality of the ring blocking the birth canal, or baby getting tangled. Other participants spoke of the ring contributing to excessive discharge and feeling heavy. The frequency of these comments and reports suggests that even those who did not voice concerns may still wonder about or have them, and study pamphlets and counseling messages could pre-emptively address frequent fears, e.g. messages to address noted fears such as “the ring will not block, choke, entangle or harm the baby during delivery”, or more simply: “the ring will not affect the baby’s safety during delivery”. Counseling and materials could additionally address the frequency with which discharge and heaviness are reported in pregnancy, and the increases in these experiences towards the later stages of pregnancy. Further, feelings of heaviness might be particularly exacerbated in subsequent pregnancies because of the laxity in the pelvic floor, not because of intravaginal ring use. Future demonstration projects and program activities with oral and vaginal PrEP available for pregnant and breastfeeding populations will offer more experiential safety and effectiveness data at the population level and will clarify what concerns persist and require provider counseling and support.

Overall, these late-stage pregnant participants, like their nonpregnant counterparts in previous trials, found the ring and oral PrEP, albeit with some misgivings, acceptable [3–5]. Our analysis of acceptability used a theoretical framework called the TFA that was originally designed to assess seven components of acceptability of behavioral and psychotherapy interventions (Fig. 1). The benefit of using this approach and framework to measure acceptability of a biomedical HIV prevention method was that the concept of “acceptability” is objectively broad, and not universally defined. Application of the TFA during qualitative analysis helped to ensure that potentially relevant acceptability sub-components that might otherwise have been overlooked or under-measured, and that were representative of an individual’s belief system, knowledge, attitudes and experiences (and other factors), were considered. A measurement tool to quantitatively assess acceptability and the constructs of this framework has recently been published [29]. Qualitative research is, and should be, inherently iterative and open-ended, however development of interview guides can thoughtfully consider inclusion of questions and probes that explore framework constructs.

There are several potential limitations to this paper that should be considered. The cohort 1 pregnant people who joined the MTN-042/DELIVER study at late-stage pregnancy may have different attitudes than other pregnant people (irrespective of gestational age), and other non-pregnant people. For example, they may be more motivated to join a research study that offers access to high-quality healthcare services and HIV prevention for themselves and their babies at a timepoint near delivery. Enrolment into the first late-stage pregnancy cohort also meant that follow-up time was brief. Product use was therefore limited in time and attitudes may change for those who use PrEP for a longer period. Nevertheless, many of the key acceptability attitudes aligned with those reported during formative work, a period of time defined as “prospective acceptability” where participants have not yet engaged in an intervention (e.g. used the ring and oral PrEP, see Fig. 1). Additional research with earlier gestational age cohorts will elucidate if attitudes persist or change with longer duration of product experience. Also because of the late stage of pregnancy, attitudes and experiences were captured within a timeframe defined by a physically heavy and uncomfortable period of pregnancy, which may have negatively impacted acceptability of an intravaginal product. Interviews were also conducted during a period of COVID-19 restrictions where masks or other factors may have interfered with the interview, affecting data clarity and collection processes. There are potential biases introduced by participants perceiving a need to report socially desirable attitudes about study products, however here several participants were candid about their concerns. There may be errors introduced by inaccurate interpretation or understanding of qualitative data through text translation or -etic (outsider) perspectives. Transcription and translation QC procedures and results interpretation through collaboration with site-based scientists aimed to minimize misinterpretation.

In conclusion, the data captured from this study confirm both positive and negative perceptions about the impact of the ring and oral PrEP use on people late in pregnancy and unborn babies, and many could be assuaged or clarified (as appropriate) with provider support. Clinic staff support and counseling about commonly-documented concerns, including any related to product-use disclosure and engagement of partners should form an essential component of ring and oral PrEP rollout among pregnant people.
